# Role of Exosomes in Chronic Liver Disease Development and Their Potential Clinical Applications

**DOI:** 10.1155/2022/1695802

**Published:** 2022-05-06

**Authors:** Chen Wang, Jinwen Liu, Yongmin Yan, Youwen Tan

**Affiliations:** ^1^The Third Hospital of Zhenjiang Affiliated Jiangsu University, Jiangsu University, Zhenjiang, 212005 Jiangsu, China; ^2^School of Medicine, Jiangsu University, Zhenjiang, 212013 Jiangsu, China

## Abstract

Extracellular vesicles (EVs) are vesicular bodies (40-1000 nm) with double-layer membrane structures released by different cell types into extracellular environments, including apoptosis bodies, microvesicles, and exosomes. Exosomes (30-100 nm) are vesicles enclosed by extracellular membrane and contain effective molecules of secretory cells. They are derived from intracellular multivesicular bodies (MVBs) that fuse with the plasma membrane and release their intracellular vesicles by exocytosis. Research has shown that almost all human cells could secrete exosomes, which have a certain relationship with corresponding diseases. In chronic liver diseases, exosomes release a variety of bioactive components into extracellular spaces, mediating intercellular signal transduction and materials transport. Moreover, exosomes play a role in the diagnosis, treatment, and prognosis of various chronic liver diseases as potential biomarkers and therapeutic targets. Previous studies have found that mesenchymal stem cell-derived exosomes (MSC-ex) could alleviate acute and chronic liver injury and have the advantages of high biocompatibility and low immunogenicity. In this paper, we briefly summarize the role of exosomes in the pathogenesis of different chronic liver diseases and the latest research progresses of MSC-ex as the clinical therapeutic targets.

## 1. Introduction

Liver, as an essential metabolic organ of the body, plays an important physiological role in metabolizing toxic substances, storing liver sugar, and synthesizing secretory proteins. Chronic liver disease has become a serious public health problem with 2 million deaths worldwide per year. Minimally invasive liver biopsy remains the preferred method for pathological examination of liver disease [1], but its reliability depends on high sampling accuracy, and it is urgent to establish more reliable and noninvasive diagnostic methods to satisfy clinical demand.

The study of exosomes in liver diseases is a rapidly developing field. As the investigation develops in-depth, people have realized the critical role of exosomes in regulating cellular signal transduction and material transportation and focused on studying exosome-mediated chronic liver diseases. Exosomes from different cell types have different compositions (such as RNAs, proteins, and lipids) and thus have complex biological functions. The hepatocytes [2], Kupffer cells [3], hepatic stellate cells [4], and hepatic sinusoidal endothelial cells [5] that constitute the liver could all secrete or serve as target cells of exosomes, and the changes of the number and composition of these vesicles reflect the physiological and pathological state of liver. The quality and quantity of these exosomes vary from different liver states [6]. During the occurrence and development of liver diseases, exosomes can also be used as novel molecular biomarkers for monitoring the therapeutic effect of diseases.

Mesenchymal stem cells (MSCs) are kind of cells with the potential of self-renewal and multidifferentiation, which can be obtained from various human tissues. Exosomes derived from MSCs (MSC-ex) are tiny vesicles that carry and maintain the proteins and mRNA activity of secretory cells. As a natural cell-derived nanocarrier, MSC-ex have been reported to cross the blood-brain barrier [7]. They can directly enter the target cells to play biological roles, with higher biosafety and more stable signal transduction efficiency over parental MSCs. Therefore, MSC-ex have attracted widespread attention in the field of tissue regeneration and repair [8]. For example, some scholars have found that human periapical cyst-MSCs can differentiate into dopaminergic neurons under certain conditions, and the difference between exosomes released during this process helps people better understand the relationship between the onset of Parkinson's disease and circadian rhythm [9]. In the model of liver ischemia-reperfusion with partial hepatectomy in miniature pigs, adipose-derived stem cells (ADSCs) contributed to the repair and regenerate of damaged tissues [10]. Moreover, hucMSC-ex hold the tremendous potential in endometrial stromal cell repair, which could be used in the treatment of endometrial injury [11].

In the field of chronic liver diseases, exosomes secreted by various types of MSCs play a significant role in repairing injured tissues and regulating immune responses. This review will describe the role of exosome-mediated intercellular communication in various types of chronic liver diseases, with special emphasis on the application of MSC-ex in clinical therapy.

## 2. Role of Exosome in the Development of Chronic Liver Diseases

### 2.1. Liver Fibrosis

Liver fibrosis is a tissue damage repair reaction characterized by abnormal proliferation of intrahepatic connective tissue, which is a common pathological process in the initial stage of various chronic liver diseases, such as viral hepatitis, alcoholic fatty liver, and hepatocellular carcinoma. Liver fibrosis is the precursor to cirrhosis [12], which has been widely accepted that cirrhosis developed from hepatic fibrosis is a precancerous lesion.

In the course of hepatic fibrosis, the communication between hepatic stellate cells and other hepatic parenchymal cells such as hepatic sinusoidal endothelial cells and Kupffer cells plays an important role in the occurrence and development of hepatic fibrosis. Exosomes released by damaged hepatocytes internalize in hepatic stellate cells, leading to phenotypic switch of quiescent stellate cells. The activation of hepatic stellate cells (HSCs) is the primary driving factor to the occurrence, development, and regression of liver fibrosis [4]. Exosomes released by damaged hepatic stellate cells are rich in fibrogenic contents, which can promote fibrosis through multiple pathways [13], for example, stimulating collagen production by fibroblasts, myofibroblasts from bone marrow, and portal fibrocytes [14]. Charrier et al. found the connective tissue growth factor (CTGF), a multifunctional heparin-binding glycoprotein that plays a promoting role in a variety of fibrosis processes. CTGF is widely present in activated HSC-derived exosomes [15], regulating the activation and migration of HSCs and immune responses, while EVs produced by quiescent HSCs are rich in miR-214, Twis1t, which attenuates the profibrotic function of activated HSCs. Exosomes derived from hepatic sinusoidal endothelial cells regulate the migration capacity of HSC by adhesion [16], and adhesion promotes the entry of EVs into target cells through dynein-dependent endocytosis. EVs produced by healthy human serum, such as miR-34c, miR-151-3p, miR-483-5p, and miR-532-5p [17], inhibit the activation of stellate cells and fibrogenesis pathways. Moreover, liver injury promotes the activation of monocytes and macrophages [18] and produces a series of proinflammatory mediators that cause liver inflammation through exosomes.

**Figure 1:**
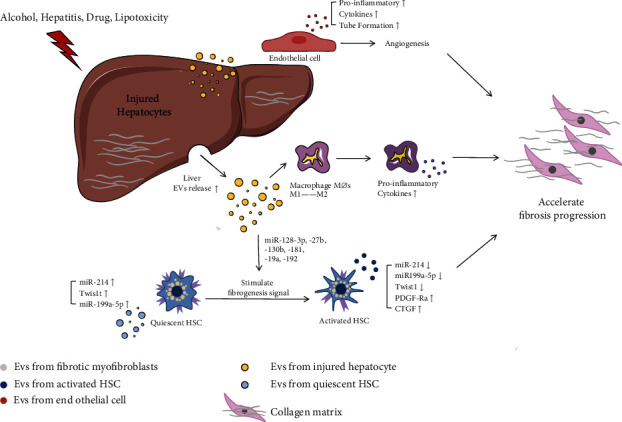
Functions of exosomes in the pathogenesis of liver fibrosis. Hepatocyte-derived exosomes activated by the exposure to alcohol, lipotoxic damage, and hepatitis virus. A variety of different pathways mediate chronic inflammation that exacerbate liver fibrosis. Immune cells secrete a large number of proinflammatory factors to promote the infiltration of inflammatory cells and aggravate liver inflammation. During liver injury, exosomes secreted by hepatocytes contain different types of RNAs, protein, and drive activation and function in hepatic stellate cells and macrophages. Meanwhile, LSEC vascularization and extracellular matrix deposition increased, resulting in fibrosis and liver dysfunction. The most significant markers of fibrosis are intrahepatic connective tissue dysplasia and massive diffuse extracellular matrix deposition. It is marked by upregulated expression of collagen, laminin, and *α*-SMA.

The formation of liver fibrosis is closely related to the progress of various liver diseases. During the formation of liver fibrosis, exosomes produced by various cells interact with each other to jointly regulate the changes of cytokines and cell populations and function as the fibrosis regulators in the occurrence and development of liver fibrosis, and the specific mechanism of their functions still needs to be further studied (Figure 1).

### 2.2. ALD and NAFLD

#### 2.2.1. Alcoholic Liver Disease

Alcoholic liver disease (ALD) is a liver pathology associated with chronic alcohol consumption [19]. During ALD, the release of circulating exosomes increased, including specific proteome, miRNA, and lipid, which may be associated with cellular stress responses. By comparing EV components in plasma of healthy controls and patients with severe alcoholism, alcoholic cirrhosis, and alcoholic hepatitis, it was found that EV levels increased in patients with ALD and correlated with disease severity [20]. High EV levels predict poor prognosis in ALD patients. Regular consumption of alcohol causes inflammatory stimulation by inhibiting fatty acid oxidation, upregulating adipogenic genes, and changing lipid transportation.

**Figure 2:**
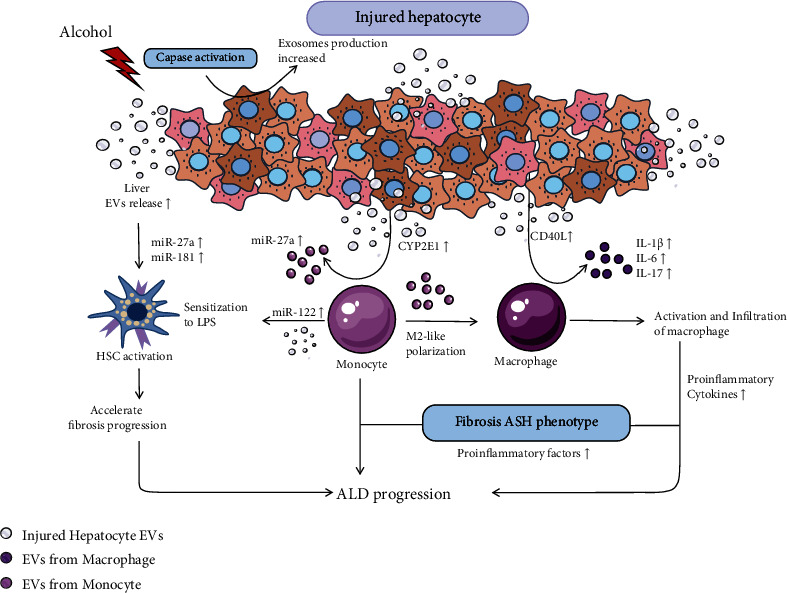
Exosomes in progression of alcoholic liver disease (ALD). Circulating exosomes play a significant role in lipid and cholesterol metabolism in alcoholic liver disease. During the progression of alcoholic liver disease, many pathways lead to the increased of exosome release. These exosomes activate hepatic stellate cells and promote fibrotic deposition. They are absorbed by immune cells such as monocytes and macrophages. Ultimately, a large number of proinflammatory factors are released, promoting the transformation of AFL into ASH.

Alcohol activates cellular regulatory networks that control inflammation and cell death, including the caspase path, which leads to the activation of apoptotic pathways and increases exosomes' production [21]. Alcohol-mediated release of exosomes is rich in CYP2E1 [22] from hepatocytes. CYP2E1 is a member of the cytochrome P450 family, promoting monocyte polarization that secretes exosomes with high expression of miR-27a. Alcohol also inhibits the phosphorylation of JNK and P38 by MAP2K4, MAP2K7, and p38 MAPK pathways and activates ERK, leading to the increased secretion of IL-10 in monocytes. IL-10 is a differentiating factor that induces the generation of M2-polarized macrophages [23]. Alcohol-induced hepatocytes secrete exosomes with high expression of CD40L and miR-122 [24]. Monocytes increased their sensitivity to lipopolysaccharides [25] after receiving exosomes rich in miR-122, and macrophages were activated and released many proinflammatory factors like IL-6, IL-17, and IL-1*β* after receiving CD40L [26]. During the progression of ALD, the toll-like receptor 4 (TLR4) pathway is activated and induces toll-receptor ligands binding to miR-155, triggering inflammation and liver injury. Hepatocyte-derived and monocyte-derived exosomes both regulate macrophage phenotypes, leading to the inflammatory phenotype of ALD. In alcoholic steatohepatitis, exosomes released by intestinal epithelial cells in the enterohepatic circulation had an adverse effect on hepatocyte activity and lipid accumulation [27], driving infiltration of macrophages and neutrophils [19]. In summary, these bioactive molecules released by exosomes mediate intercellular signal transduction and suggest the progression of ALD to alcoholic fatty liver (Figure 2). In addition, exosomes are widely present in body fluids and carry alcohol-related specific components, which can also be used as markers for the diagnosis of alcoholic liver injury, but their specificity and sensitivity need to be evaluated.

#### 2.2.2. Nonalcoholic Fatty Liver Disease

Nonalcoholic fatty liver disease (NAFLD) starts with steatosis and progresses to nonalcoholic steatohepatitis (NASH), which is one of the most common chronic liver diseases associated with obesity, insulin resistance, and genetic susceptibility [28] and has the risk of developing into terminal liver diseases [29]. Studies have shown that 20% of NAFLD patients could advance to NASH and eventually cirrhosis [30]. Exosomes play an essential role in the pathogenesis of NAFLD.

NAFLD is characterized by hepatocyte dysfunction induced by lipid and macrophage-associated inflammation [31]. During the development of NAFLD, lipid toxicity signals promote monocytes to the liver and polarize into inflammatory macrophages [32]; the lipid-induced death receptor five signaling pathway is activated, and the damaged or stressed hepatocytes release exosomes that are closely related to the degree of liver injury [33]. Diet-related steatohepatitis affects exosome release and some obesity-related exosomes, such as miR-27A-3p, miR-27b-3p, miR-192, and miR-122 [34], overexpressed in circulating exosomes isolated from high-fat-fed mice. miR-199a-5p in circulating exosomes of high-fat-fed mice inhibits macrophage stimulation and fatty acid metabolism, thus promoting lipid accumulation in the liver [35]. In addition, lipids cause the accumulation of immature bone marrow cells, which release proinflammatory cytokines and induce apoptosis of natural killer T (NKT) cells. NKT apoptosis promotes the excessive production of TH-1 cytokines, leading to chronic inflammation. Lipotoxic exosomes further induce angiogenesis through vascular noninflammatory protein-1 and mediate its internalization by endothelial cells. Lipotoxic hepatocytes also produce exosomes rich in miR-17-92 clusters, absorbed by HSCs, leading to fibrotic activation [36].

**Figure 3:**
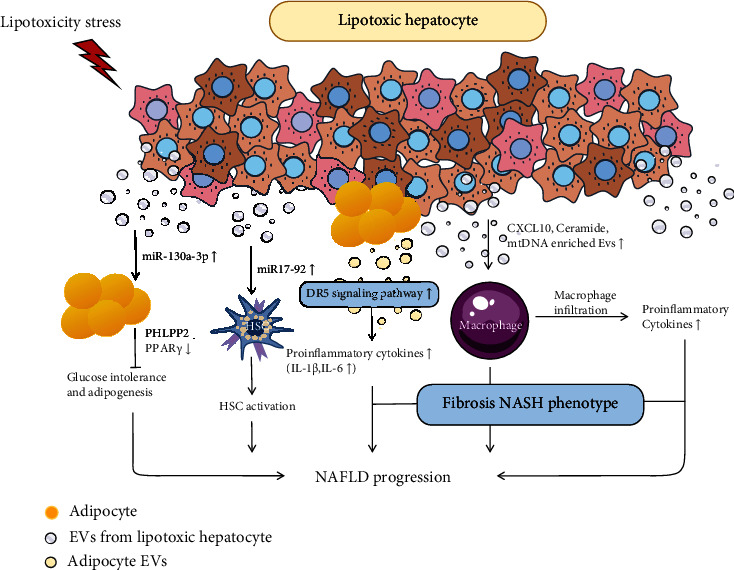
Exosomes in progression of alcohol-associated liver disease (NAFLD). In the progression of NAFLD, various lipotoxic stimuli promote hepatocyte steatosis and promote the release of hepatocyte-derived exosomes. The exosomes released carry components composed of proteins, RNAs, and lipids. Exosomes promote the progression of NAFLD. On the one hand, it facilitates the infiltration of immune cells in liver and promotes liver inflammation; on the other hand, it participates in insulin resistance and activates stellate cells.

The progression of NAFLD to NASH depends on the exosome-mediated cell-to-cell communications. After the passage of NAFLD into NASH, exosomes of hepatocytes rich in mtDNA are released. The mtDNA-rich exosomes activate TLR9 in KCs by triggering the secretion of proinflammatory cytokines, such as IL-1b and TNF-*α*, and aggravate the progression of NAFLD [37]. Interestingly, exosomes from adipose stem cells reduce adipose inflammation and lipid deposition by polarizing M2 macrophages and white adipose tissue. Adipose tissue is an essential source of circulating miRNA, and adipose cells with Dicer specifically knocking out significantly reduce the numbers of circulating miRNA [38]. In addition, HepG2-derived exosomes can be actively internalized by adipocytes, thus stimulating transcriptome changes in adipocytes, specifically inducing inflammatory phenotypes in adipocytes [39]. These findings suggest that the secretion of vesicles containing unique substances may trigger the progression of NAFLD to other liver diseases by inducing the activation of macrophages and the release of inflammatory factors (Figure 3).

In summary, during the occurrence and development of alcoholic and nonalcoholic liver diseases, exosomes regulate the signal transduction and materials transfer between hepatocytes and inflammatory cells, affecting the activities of the liver mononuclear macrophage system and regulating the inflammatory responses.

### 2.3. Viral Hepatitis

There are about 1.5 million people worldwide die of hepatitis virus-related liver diseases every year [40], creating a serious public health problem. Persistent viral replication and low immunologic function result in severe liver parenchymal damage and increase the risk of viral hepatitis progression to terminal liver disease. Here we mainly introduce the application of exosomes in viral hepatitis B and C.

Exosomes are potent vectors for transmitting the hepatitis virus and transmitting nucleic acids and proteins of the virus from infected cells to uninfected cells. Exosomes secreted by hepatocytes infected with hepatitis C virus carry virus-derived Ago2 protein, HSP90, and miR-122 [41], mediating stable transmission of hepatitis virus in the liver [42]. On the one hand, exosome-mediated viral transportations help the virus evade surveillance by the immune system. miRNAs released from virus-infected hepatocytes inhibit the proliferation and survival of natural killer (NK) cells and escape the host's innate immunity [43]. Exosomes containing HCV RNA reduce toll-like receptor 3 (TLR3) activation and interfere with antiviral ISG activation [44]. The expression of TIM-3/GAL-9 in exosomes secreted by HCV-infected hepatocytes increased, affecting the differentiation of monocytes and suppressing the body's immune responses [45]. The complex microenvironments in the liver, on the other hand, could also identify particles carrying viral antigens, inducing the immune responses of target cells to the virus [46]. Innate immune responses to the virus removal depend on NK cells, dendritic cells (DCs), and T cells. Exosomes released from immune cells secrete the antiviral factors and immunosuppressive factors. NK cells also secrete exosomes with the natural killing ability and antiviral proteins, such as CD56 and perforin. DC is the most effective antigen-presenting cell against hepatitis virus attacks. Macrophage-derived exosomes transfer antiviral responses induced by interferon-*α* (IFN-*α*) could be transmitted from liver nonsubstantial cells [47] and macrophages [48] to HBV-infected hepatocytes via exosomes to exert the function of antivirus. In addition, exosomes released by hepatocytes infected with HBV also carry HSP70 protein [49] and stimulate macrophages to express the NK cell-active receptor ligands through the signal transduction pathway, thus promoting the antiviral ability of NK cells. In some nonparenchymal cells in the liver, although hepatitis virus does not replicate in these cells, they still induce intracellular expression of cytokines like interferon I and III types that stimulate the immune system and exert an antiviral response release exosomes into virus-infected hepatocytes [47]. Interestingly, blocking the release of EVs severely inhibits viral replication but without suppressing the viability of host cells [50]. These studies have proved that exosomes play an essential role in virus transmission and immune regulation.

**Figure 4:**
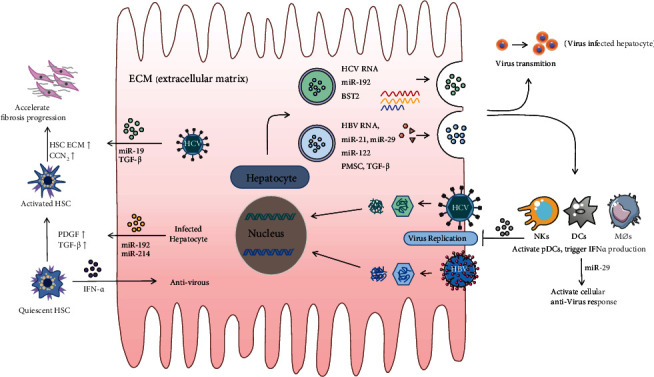
The schematic represents exosomes in viral hepatitis and their molecular mechanisms by which exosomes regulate cellular communication in liver. As indicated in the figure, the hepatitis B virus (HBV) and hepatitis C virus (HCV) rely on exosomes to spread viral RNAs and protein to adjacent liver cells, and these particles facilitate the spread of the virus as well as destroy the innate immune response of the host.

The regulation of exosome-mediated intercellular communications might be an effective method to control hepatitis virus transmission. Evidence has shown that exosomes released by hepatocytes during viral hepatitis are involved in the HSC-mediated liver fibrosis pathway. HCV-infected hepatocytes release exosomes enriched in miR-192 and miR-214 and deliver them to HSCs, resulting in HSC activation and transformation into myofibroblasts that highly express fibrotic components such as CNN2 [51]. HCV-infected hepatocytes could also release exosomes expressing miR-19a, which directly regulate the CSS-STAT3 axis and upregulate extracellular matrix (ECM) factors in HSC [52] and activate the profibrosis pathway of HSC through exosome-mediated autocrine [53]. In summary, the role of exosomes in viral hepatitis is complex, and the inhibition of the EV release may play an antiviral effect to a certain extent. Further animal experiments and preclinical studies for the function of exosomes will help to predict the prognosis of the diseases and develop new therapeutic strategies (Figure 4).

### 2.4. Hepatocellular Carcinoma

Hepatocellular carcinoma (HCC) is a common malignancy with a poor global survival rate. The main risk factors of HCC encompass viral hepatitis infection, excessive alcohol consumption, and smoking [54], but it is complicated and difficult to screen the pathogenesis of HCC.

Plentiful evidences suggest that exosomes derived from hepatoma carcinoma cells carry tumor-specific markers, which mediate the intercellular communication between cancer cell populations, and promote the migration and invasion of recipient cells. It was found that the expression of circ-0004277 in exosomes from hepatoma carcinoma cells upregulated and improved the expression of circ-0004277 in normal hepatocytes adjacent to hepatoma carcinoma cells through cell communications thus inducing epithelial-mesenchymal transformation (EMT) and promoting intrahepatic metastasis of HCC [55]. EMT is the key process of tumor metastasis [56], and tumor cells receiving EMT can release exosomes conducive to tumor metastasis [57]. High-throughput sequencing revealed that miR-374A-5p has the most significant differential expression among miRNA components in exosomes [57]. Recent studies also show that hepatoma carcinoma cells cultured with cancer-derived exosomes increase the number of cancer stem cells. Hepatoma carcinoma cells secrete exosomes that highly express Shh and activate the hedgehog pathway to promote tumorigenesis [58]. These results suggest that hepatoma carcinoma cells mediate the secretion of different exosomes, regulate the liver microenvironments through various pathways, and promote tumor migration and invasion.

**Figure 5:**
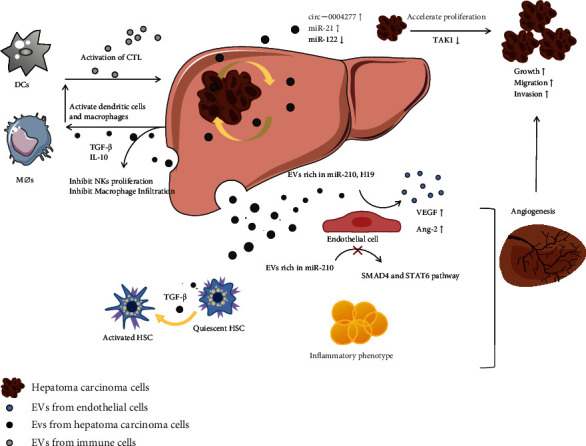
The detailed mechanism of exosomes in hepatocellular carcinoma (HCC). Hepatoma carcinoma cells influence neighboring cells through exosomes and established tumorigenic microenvironment. On one hand, cancer cell-derived exosomes promote diffusion of tumor contents to normal cells, promote epithelial-mesenchymal transformation and tumor angiogenesis, and promote stellate cell activation that is related to liver fibrosis pathway. On the other hand, cancer cell-derived exosomes could present and deliver relevant information to activate the immune responses of the body or inhibit immune cell function to promote tumor immune-escape.

In addition, the recurrence and metastasis of hepatocellular carcinoma can seriously affect the therapeutic effect of the disease. It is well known that hypoxia plays a key role in the progression of HCC [59]. Hypoxia increases the production of exosomes by HCC cells [60]. In a study of hypoxia-induced exosome expression, scholars found that exosomes released by HCC cells can activate the Wnt/*β*-catenin signaling pathway through the expression of miR-1273F and enhance malignant phenotype [60] like migration, proliferation, EMT, and invasion of normoxic HCC cells. A recent study showed that hypoxic environment promotes exosome release in CRC (colorectal cancer, CRC) lesions, and miR-135a-5p components in exosomes may be involved in an important component of colorectal liver metastases [61]. We have reasons to believe that exosomes play an integral role in hypoxia-induced tumor metastasis. Moreover, highly angiogenic microenvironment provides sufficient nutrients for tumor cell growth [62]. On the one hand, exosomes from hepatocellular carcinoma cells, like miR-103, regulate the biology of vascular endothelial cells to inhibit the synthesis of VE-cadherin and P120 connexins [63], thereby increase vascular barrier permeability and promote the metastasis of HCC. On the other hand, exosomes promote tumor angiogenesis. miR-378b-riched exosomes from hepatoma carcinoma cells enhance the angiogenesis of HCC [64], which may be associated with TGFBR3 inhibition. Exosomes derived from hepatoma carcinoma cells are also rich in miR-210 and promote angiogenesis in vitro and in vivo that significantly correlated with blocking SMAD4 and STAT6 pathways [65]. These results suggest that malignant tumor cells could alter the tumor microenvironments through exosomes and enhance the degree of angiogenesis in HCC (Figure 5).

## 3. Exosomes as Biomarkers for Liver Diseases

Early detection of liver diseases is one of the essential prerequisites for blocking the disease progression. Invasive liver biopsy remains the reference standard for diagnosing liver diseases, and novel, reliable, and noninvasive diagnostic methods are urgently needed. Exosomes from a variety of human tissues such as urine and blood are currently considered as source of noninvasive molecular biomarkers for early detection and prognosis of various liver diseases. The components of exosomes such as particular proteins and nucleic acids can cross the blood-brain barrier, representing the physiological and pathological states of the liver.

Exosomes play a key role in mediating cell to cell communication and package delivery, and they can be used as diagnostic biomarkers. It was found that dozens of miRNAs significantly increased in the serum of HCC patients compared with healthy controls. Among them, miR-210 mediated by exosomes derived from hepatoma carcinoma cells increased and could be used as a biological marker for diagnosing HCC [65]. miR-638 in serum exosomes affects the occurrence of HCC by inhibiting the proliferation of cancer cells and is considered a tumor-specific miRNA marker. In patients with chronic hepatitis B, viral load is proportional to serum miR-122 level, and miR-146a level has an opposite trend, both involved in inflammatory and immune responses [66]. Low levels of serum miR-122 may indicate severe liver fibrosis. And the elevation of miR-21 suggests HBV-associated cirrhosis or HCC [67]. miR-19a and miR-155 levels are associated with advanced liver fibrosis in the serum exosomes of HCV patients, and the CD81 protein content in serum exosome was positively associated with inflammatory activity and severity of liver fibrosis [68]. Sphingolipids in plasma-derived EVs could be used as biomarkers in patients with alcoholic fatty liver disease. The impurities from lipoprotein have little interference, showing a high diagnostic value [69]. For example, miR-309, miR-30a, and miR-192 increased significantly in alcohol-induced liver injury, which has a high diagnostic value for the identification [20]. In patients with NAFLD, lipotoxic hepatocytes release exosomes highly expressing MLK3, and multiple miRNAs in serum of patients, such as miR-34a, miR-122, and miR-192, could be used as biomarkers of NAFLD [70] (Table 1).

Liver disease is a worldwide difficult problem. As part of liquid biopsy, exosomes are expected to be used in the early diagnosis of chronic liver disease. As a new generation of nanomedical diagnosis, people expect to build a nanodiagnostic platform based on EVs. Although further studies are needed to improve the specificity of exosome in various chronic liver diseases, we believe that exosomes as novel biomarkers have widespread clinical application value. It is necessary to combine multiple indicators as specific markers for early diagnosis and monitoring liver diseases to pursue unsatisfied clinical needs.

## 4. MSC-ex Exert Applications in the Treatments of Liver Diseases

Liver transplantation (LT) remains the standard treatment for almost all the end-stage liver diseases (ESLDs), but the imbalance between donors and the patients and the complex postoperative complications are the major challenges. There are few specific targeted drugs for liver diseases clinically at present, and it is urgent to develop new drugs for ESLD.

Mesenchymal stem cells (MSCSs) are pluripotent stem cells that derived from mesoderm and widely distributed in almost all the body tissues. Study has found that the proteins and miRNAs contained in mesenchymal stem cells (MSCs) play their functions in the pathological process of liver and regulate liver microenvironment. Exosomes are small vesicles secreted by cells. Compared with mesenchymal stem cells (MSCs), the exosomes secreted by MSCs are smaller and lower immunogenicity. They are easier to produce and store and even easier to avoid formation of ectopic tissue or tumor masses and avoid some of the regulatory issues that allogeneic mesenchymal stem cells face. Therefore, the transplantation of MSC-ex has become the focus of injury repair and regenerative medicine. Studies have found that exosomes could pass through the intercellular spaces and deliver “therapeutic molecules” between different cells in chronic liver diseases. In tumor microenvironment, MSC-ex could transmit extracellular signals and inhibit tumor angiogenesis by downregulation of vascular endothelial growth factor (VEGF) [104]. hucMSC-ex could transfer bioactive components and reduce carbon tetrachloride- (CCl4-) induced mouse liver fibrosis [105], inhibiting EMT and collagen production and upregulating the expression of apoptotic protein Bcl-2 in hepatocytes. Correspondingly, exosomes from amnion-derived MSCs (Ad-MSCs) attenuate CCl4-induced liver injury by inhibiting stellate cells and Kupffer cell activation [106]. Exosomes from human-induced pluripotent stem cells (IPSCs) promote regeneration against the ischemia/reperfusion model in mice [107]. Moreover, exosomes derived from BM-MSCs could suppress the Wnt/*β*-catenin pathway axis to improve liver fibrosis [108]. In addition, exosomes from healthy people may benefit patients with liver fibrosis. For example, exosomes from normal hepatocytes reduce the expression of fibrosis-related genes in mice induced by CCl4. Exosomes derived from human hepatocytes indicate an excellent ability of antifibrosis and anti-inflammatory in the NASH model of mice, inhibiting HCC growth and stimulating its apoptosis by the delivery of miR-451 and miR-31 [109]. These studies provide a theoretical basis for the potential therapeutic effects of exosomes in liver diseases (Table 2).

Recent research has suggested that exosomes contain organ-specific targeting molecules specific to recipient tissues to some extent, and gene modification can improve the therapeutic effect of MSC exosomes. In addition to the direct therapeutic effects of exosomes on chronic liver diseases, exosomes can also be used as vectors to deliver specific biomolecules to target cells for biological functions, with the development of nanotechnology and its deepening in the interdisciplinary fields like biomedicine. Nanovesicles, traditionally used as a single drug carrier, have been endowed with a variety of new functions, while their activity in the body has been improved. Cell-derived exosomes are a kind of endogenous nanodrug treatment system based on cell derivatives. Compared with traditional synthetic nanocarriers prepared in chemical environment, they have excellent biocompatibility and bioavailability. How to give full play to the advantages of nanomaterials and at the same time to synthesize or extract innovative nanodrugs with low toxicity as diagnosis tracer or treatment of diseases has become the frontier of the development of nanomaterials. Naturally occurring exosomes have the disadvantages of short half-life and fast clearance rate. Recent studies have shown that modified exosomes or hydrogel-coated exosomes can prolong the retention rate in vivo and improve the therapeutic effect. In addition, it has been suggested that the efficacy of MSC-ex may depend on its biological composition and, more importantly, on the responsiveness of the recipient to MSC-ex [110]. Some substances in the environment of MSC-ex may also be important components that inhibit its activity. In the treatment of peri-implantitis in dental implant treatments with MSC-ex, scholars have found the phenomenon of oxidative stress and vessel morphology alterations under the exposure of titanium (Ti) particle, which influence the function of MSC-ex [111].

In addition, quantitative criteria for exosome as a treatment means should be established, and various clinical trials are needed to determine its safety, efficacy, and feasibility in the body. With the progress of science and technology, the methods of exosome extraction are changing rapidly; however, there is no unified extraction scheme for exosomes to date.

## 5. Conclusion and Prospect

This review summarizes the role of MSC-ex in alleviating the progression of chronic liver diseases and the value of exosomes as potential diagnostic and therapeutic markers in chronic liver diseases. Exosomes are closely connected with chronic liver diseases, and the change of their composition may reflect the underlying disease progression. The release of exosomes increased in various chronic liver diseases, and these small vesicles could carry molecules with biological activity such as proteins, nucleic acids, and lipids for relatively stable transmission between cells and participate in a variety of pathophysiological processes, such as cytokine secretion, macrophage activation, extracellular matrix remodeling, and hepatic stellate cell activation.

The characteristics of exosomes carrying small molecules and the biological activity determine their potential role in the treatment of liver diseases. Many studies have been carried out in chronic liver diseases and have made some progress. However, exosomes are as fraught with expectations as they are with problems. Most of the current studies on exosomes for the treatment of chronic liver diseases are based on cell experiments and animal experiments, and the clinical phase of the investigation remains to be explored. Moreover, for EVs in circulating body fluid or blood, exosomes and microvesicles are defined by size, but they cannot be distinguished by size. Therefore, it becomes especially important to distinguish circulating EVs by expression of specific biomarkers in exosomes and microvesicles. We also need to further explore the precise molecular mechanisms of exosome biogenesis, release, and interaction with target cells in chronic liver diseases for clinical transformation and define the criteria for biological properties of exosomes during mass production. With the participation of a growing number of scholars, it is believed that exosomes will be widely used in clinical practice in the near future, bringing good news to patients.

## Figures and Tables

**Table 1:** Exosomes as possible biomarkers of different liver diseases.

Disease	Source	Content	Expression	Clinical significance	Refs.
Liver fibrosis	Serum	miR-21, miR-122, miR-155, miR-214	Up	Detection, progression, and diagnosis of fibrogenesis	[20, 71–73]
		miR-122	Down	Severe liver fibrosis; suggest NASH-induced liver fibrosis	[74]
		miR-199a		Significantly decreased in patients with cirrhosis and HCC	[75]

ALD	Serum/plasma	miR-122, miR-155, miR-146, miR-192, miR-30a, miR-340, miR-744	Up	Differential diagnosis and progression of ALD	[76, 77, 20]

NAFLD	Serum	miR-21, miR-34a, miR-192	Up	Potential diagnostic biomarker and forecast progression	[70, 78, 79]
		miR-122,	Up	An attractive therapeutic/the target of lipid metabolism	[80]
	Hepatocytes	MLK3	Up	A potential diagnostic biomarker of NAFLD	[81]

Viral hepatitis	Serum	miR-21, miR-19a	Up	A potential novel biomarker for diagnosis	[82, 83]
		miR-483-5p, miR-672-5p	Up	Progression of liver fibrosis in CHC	[84]
	Serum	miR-122, Ago2, HSP90	Up	Enhance HCV replication	[41]
	Macrophage	miR-29	Up	HCV infection (in vitro)	[85]
	Serum	miR-106b, miR-1274a, miR-130a, miR-140-3p, miR-151, miR-3p, miR-181a, miR-21, miR-24, miR-375, miR-93	Down	Progression of liver fibrosis in CHC	[84]
	Plasma	miR-150, -192, -200b, -92a	Down	Decreased in HBV or HCV patients	[86]

HCC	Serum	LINC00161	Up	Predict tumor growth and metastasis in HCC	[87]
		miR-21, miR-92b, miR-93, miR-665, miR-155	Up	Detection, prognosis, and recurrence of HCC	[88–92]
		miR-1247-5p, miR-224, miR-210-3p	Up	Detection, diagnosis, and therapeutic target	[93, 94] [65]
		miR-638	Up	Specific and promising for surveillance marker	[95]
		miR-519d, miR-494		Independent diagnostic biomarkers	[96]
	Serum	miR-744, miR-9-3pmi-125b, miR-718	Down	Detection, prognosis, recurrence, and therapeutic target of HCC	[96–99]
		miR-638, miR-122, miR-195	Down	Detection, prognosis, and recurrence of HCC	[100–102]
	Plasma	miR-92a-3p	Up	Potential diagnostic biomarker of HCC	[103]

Up: upregulated; down: downregulated; HCC: hepatocellular carcinoma; ALD: alcoholic liver disease; NAFLD: nonalcoholic fatty liver disease.

**Table 2:** Therapeutic effects of MSC-derived exosomes in liver diseases.

Source	Active ingredients	Models	Function	Refs.
HiPSC-MSCs	—	Ischemia-reperfusion/liver injury	Protects the liver from ischemia-reperfusion injury	[112]

Ad-MSCs	miRNA-181-5p	CCl4/mouse liver injury	Activates autophagy to prevent liver fibrosis	[113]
	miR-122	HepG2 mice transplanted with tumor	Enhance chemotherapeutic sensitivity of liver cancer	[114]
	miR-122	CCl4/mouse liver fibrosis	Inhibits collagen synthesis and stellate cell proliferation	[115]
	miR-17	GalN/TNF-*α* induced mouse ALF models	Targeting TXNIP and reducing inflammasome activation of NLRP3 in macrophages	[116]

HUC-MSCs	—	CCl4/mouse liver fibrosis	Inhibits hepatocyte EMT and collagen production	[105]
	GPX1	CCl4/liver failure	Inhibits oxidative stress of hepatocytes	[117]
	miR-451a	EMT of hepatocellular carcinoma cells	Inhibits ADAM10 and suppresses the paclitaxel resistance, cell cycle transition, proliferation, migration, and invasion	[118]
	miR-455-3p	IL-6/acute liver injury	Attenuates macrophage infiltration and reduces inflammatory factors in serum	[119]
	—	ALF	Inhibits NLRP3 activation in macrophage and decreases proinflammatory cytokine level	[120]

PL-MSCs	—	ALF	The upregulated CRP participates in vascular remodeling and promotes liver regeneration	[121]

ESC-MSCs	—	CCl4/acute liver injury	Promotes liver cell proliferation	[122]

BM-MSCs	—	Con-a/mouse liver injury	Inhibits HSC activation	[108]
	IL-6	NAFLD	Macrophages transfer antifibrotic miR-223-enriched exosomes into hepatocytes	[123]
	miR-92b-3p, miR-23b-3p, miR-204-3p, miR-1247-3p, miR-326-5p	CCl4/acute liver injury	Upregulates TNF-stimulated gene 6 and/or represses mitochondrial oxidative phosphorylation	[124]

CP-MSCs	miR-125b	CCl4/rat liver fibrosis	Promotes liver regeneration and inhibits HSC activation	[125]

HiPSC-MSCs: MSCs derived from human-induced pluripotent stem cells; Ad-MSCs: adipose-derived MSCs; HUC-MSCs: human umbilical cord MSCs; PL-MSCs: placenta-derived MSCs; ESC-MSCs: embryonic stem cell-derived MSCs; BM-MSCs: bone marrow-derived MSCs; CP-MSCs: chorionic plate-derived MSCs.

## Abbreviations

ADSC: Adipose-derived stem cell
Ad-MSC: Adipose-derived MSC
ALD: Alcoholic liver disease
ALF: Acute liver failure
BM-MSC: Bone marrow-derived MSC
CCl4: Carbon tetrachloride
CRC: Colorectal cancer
CRP: C-reactive protein
CTGF: Connective tissue growth factor
DC: Dendritic cell
ECM: Extracellular matrix
EMT: Epithelial-mesenchymal transformation
ESC-MSC: Embryonic stem cell-derived MSC
ESLD: End-stage liver disease
EV: Extracellular vesicle
HBV: Hepatitis B virus
HCC: Hepatocellular carcinoma
HSC: Hepatic stellate cell
IFN-*α*: Interferon-*α*
IPSC: Induced pluripotent stem cell
LC: Liver cirrhosis
LT: Liver transplantation
miR: MicroRNA
MSC-ex: Mesenchymal stem cell-derived exosomes
MVB: Multivesicular body
NAFLD: Nonalcoholic fatty liver disease
NASH: Nonalcoholic steatohepatitis
NKT: Natural killer T
PL-MSC: Placenta-derived MSC
TLR: Toll-like receptor
TNF-*α*: Tumor necrosis factor *α*
VEGF: Vascular endothelial growth factor.
